# Supplementary material for: Assistance dogs for military veterans with PTSD: A systematic review, meta-analysis, and meta-synthesis
Source: PLoS One. 2022 Sep 21;17(9):e0274960. doi: 10.1371/journal.pone.0274960 (PMC9491613; doi:10.1371/journal.pone.0274960)
Supplement: S1 Table — ti Title. ab Abstract. Exact search syntax was adjusted based on the vocabulary for each database. (DOCX) [file pone.0274960.s001.docx]

**S1 Table** Database search vocabulary and syntax

| **ProQuest Research Library Search Syntax** |
| --- |
| ti (( "service animal*" OR "service dog*" OR "assistance animal*" OR "assistance dog*" ) AND  ( PTSD OR "Posttraumatic stress disorder" OR "Post-traumatic stress disorder" OR "Post traumatic stress disorder" )) OR ab (( "service animal*" OR "service dog*" OR "assistance animal*" OR "assistance dog*" ) AND ( PTSD OR "Posttraumatic stress disorder" OR "Post-traumatic stress disorder" OR "Post traumatic stress disorder" )) |
| ***Notes.*** *ti Title. ab Abstract. Exact search syntax was adjusted based on the vocabulary for each database.* |
