# Supplementary material for: Assistance dogs for military veterans with PTSD: A systematic review, meta-analysis, and meta-synthesis
Source: PLoS One. 2022 Sep 21;17(9):e0274960. doi: 10.1371/journal.pone.0274960 (PMC9491613; doi:10.1371/journal.pone.0274960)
Supplement: S2 Table — (DOCX) [file pone.0274960.s002.docx]

**S2 Table** Methodological Rigor Scoring Questions

| All |
| --- |
| Was an aim, purpose, objective, or research question of the study stated? |
| Is there a clear description of eligibility (inclusion/exclusion) criteria of participants? |
| Was ethical approval sought and received, and clearly stated including source? |
| Were study participant's disabilities described beyond simply PTSD, or independently assessed by researcher? |
| Were characteristics of the service animals in the study described, including their provider and training? |
| If participants have had service animals for variable amounts of time, was time since placement considered for analyses? |
| Were limitations of the study discussed in detail taking into account sources of potential bias or imprecision? |
| Quantitative |
| Was a hypothesis/hypotheses stated? |
| Are effect sizes for most outcomes provided? |
| Does the design include a control condition? |
| Does the study provide estimates of the variability in the data for most outcomes? |
| Have actual probability values been reported for most outcomes? |
| Was there a demonstration that groups or baseline characteristics were comparable on demographic and medical variables? |
| Were key demographic characteristics of study participants described including average age and percent male/female? |
| Were statistical values for most outcomes reported? |
| Qualitative |
| Are negative/discrepant results taken into account? |
| Are sequences from the original data presented and were these fairly selected? |
| Are the explanations for the results plausible and coherent? |
| Do the authors report achieving data saturation? |
| Is it clear how the themes and concepts were identified in the data? |
| Is it clear what methods were used to collect data with sufficient details, including type of method and tools? |
| Is there triangulation of data? |
| Was the analysis performed by more than one researcher? |
