# Supplementary material for: Assistance dogs for military veterans with PTSD: A systematic review, meta-analysis, and meta-synthesis
Source: PLoS One. 2022 Sep 21;17(9):e0274960. doi: 10.1371/journal.pone.0274960 (PMC9491613; doi:10.1371/journal.pone.0274960)
Supplement: S3 Table — Ordered by most recent to least recent within each group.—Not reported. ADI Assistance Dogs International. AKC CGC American Kennel Club Canine Good Citizen Test. d Day. w Week. m Month. y Year. aStatus not reported in publication, but presumed based on other publications with same organization. (DOCX) [file pone.0274960.s003.docx]

**S3 Table** Characteristics of Service Dog Organizations

| Study | Organization(s) | Status | Accreditation | Pairing Format (Length) | Team Certification |
| --- | --- | --- | --- | --- | --- |
| Peer-reviewed |  |  |  |  |  |
| Jensen 2021 | K9s For Warriors | Nonprofit | - | - | - |
| Nieforth 2021a | K9s For Warriors | Nonprofit | - | Immersive (3w) | - |
| Nieforth 2021b | K9s For Warriors | Nonprofit | - | Immersive (3w) | - |
| Rodriguez 2021 | K9s For Warriors | Nonprofit ^a^ | - | - | - |
| Williamson 2021 | Audeamus Service Dogs | - | - | Guided training (-) | -; 6-stage process |
| Galsgaard 2020 | Department of Military Psychology, Danish Defense Service Dog Program | - | - | Guided training (10m) | - |
| Husband 2020 | Audeamus Service Dogs | Nonprofit | - | - | - |
| Lessard 2020 | CARES Citadel Canine Courageous Companions (MSAR) National service dog Thames | - | - | - | - |
| Richerson 2020 | Armed Forces Foundation Auburn Research and Technology Foundation Canine Companions for Independence | Nonprofit | ADI accredited (1) Adherent to ADI standards (2) | Immersive (1-2 w) | AKC CGC ADI Public Access Test |
| Rodriguez 2020 | K9s For Warriors | Nonprofit | ADI | Immersive (3w) | ADI Public Access Test |
| Lafollette 2019 | K9s For Warriors | Nonprofit | ADI | Immersive (3w) | - |
| McLaughlin 2019 | Young Diggers Dog Squad | - | - | - | - |
| Scotland-Coogan 2019a | - | Nonprofit | - | Guided training (14w) | Final test |
| Scotland-Coogan 2019b | - | - | - | Guided training (14w) | - |
| Whitworth 2019 | -; 2 orgs | Nonprofit | - | Guided training (14w) | Final test in public |
| Crowe 2018a | Paws and Stripes | Nonprofit | - | Guided training (up to 12m; *M* 8.89m) | ADI Public Access Test |
| Crowe 2018b | Paws and Stripes | Nonprofit | - | Guided training (up to 18m; *M* 11.67m) | ADI Public Access Test |
| Lessard 2018 | CARES Citadel Canine Courageous Companions (MSAR) National service dog Thames | - | - | Varies (5d-2y) | - |
| O'Haire 2018 | K9s For Warriors | Nonprofit ^a^ | Accredited | Immersive (3w) | - |
| Rodriguez 2018 | K9s For Warriors | Nonprofit ^a^ | - | Immersive (3w) | - |
| Yarborough 2018 | Bergin University of Canine Studies Canine Assistants Joys of Living Assistance Dogs paws4people Paws Assisting Veterans | Nonprofit | - | Immersive (2w) | Public Access Test (4)  Not reported (1) |
| Kloep 2017 | This Able Veteran | Nonprofit | - | Immersive (3w) | - |
| Vincent 2017b | -; 7 orgs | - | - | - | - |
| Yarborough 2017 | Bergin University of Canine Studies Canine Assistants Joys of Living Assistance Dogs paws4people Paws Assisting Veterans | Nonprofit | ADI accredited (4) Developing own (1) | - (2w) | ADI Public Access Test |
| Dissertations |  |  |  |  |  |
| Floore-Guetschow 2020 | -; 6 orgs | - | - | - | - |
| Hansen 2019 | 29 orgs included in recruitment.  Orgs in sample not reported. | Nonprofit | - | - | - |
| Parenti 2019 | - | - | - | - | - |
| Kegel 2016 | - | - | - | - | - |
| Kopicki 2016 | - | Nonprofit | ADI | - | - |
| Brown 2015 | - | Nonprofit | - | - | - |
| Hyde 2015 | Puppies Behind Bars | - | - | Immersive (2w) | - |
| Marston 2015 | -; 2 orgs | Nonprofit | ADI | - | Public Access Test |
| Moore 2014 | - | Nonprofit | - | - (6-8w) | Public Access Test |
| Newton 2014 | - | - | - | - | - |

***Notes.*** *Ordered by most recent to least recent within each group. - Not reported. ADI Assistance Dogs International. AKC CGC American Kennel Club Canine Good Citizen Test. d Day. w Week. m Month. y Year.*

*^a^Status not reported in publication, but presumed based on other publications with same organization.*
