# Supplementary material for: Assistance dogs for military veterans with PTSD: A systematic review, meta-analysis, and meta-synthesis
Source: PLoS One. 2022 Sep 21;17(9):e0274960. doi: 10.1371/journal.pone.0274960 (PMC9491613; doi:10.1371/journal.pone.0274960)
Supplement: S4 Table — Ordered by most recent to least recent within each group.—Not reported. LR Labrador Retriever. GR Golden Retriever. LX Labrador Retriever Mix. GSD German Shepherd Dog. X Mixed breed. V Veteran (with organizational guidance). a—Trained tasks are not reported; N Trained tasks are reported, but individual PTSD-specific tasks are not described; Y Trained tasks are reported, and PTSD-specific trained tasks are described. (DOCX) [file pone.0274960.s004.docx]

**S4 Table** Characteristics of Service Dogs

| Study | Breed | | | | | | Origin | | Training (Length) | Tasks ^a^ |
| --- | --- | --- | --- | --- | --- | --- | --- | --- | --- | --- |
|  | **LR** | **GR** | **LX** | **GSD** | **X** | **Other** |  |  | |  |
| Peer-reviewed |  |  |  |  |  |  |  |  | |  |
| Jensen 2021 | - | | | | | | - | | - | - |
| Nieforth 2021a | - | | | | | | Shelter | | Org (120+ h) | N |
| Nieforth 2021b |  |  |  | ✓ |  |  | Shelter | | Org (60+ h) | Y |
| Rodriguez 2021 | ✓ | ✓ |  |  | ✓ |  | Shelter | | - | Y |
| Williamson 2021 | - | | | | | | - | | V (-) | - |
| Galsgaard 2020 | - | | | | | | - | | V (10 m) | Y |
| Husband 2020 | - | | | | | | - | | - | - |
| Lessard 2020 | - | | | | | | - | | - | - |
| Richerson 2020 | ✓ | ✓ | ✓ | ✓ |  |  | Purpose-bred | | Org (-) | Y |
| Rodriguez 2020 | ✓ |  |  | ✓ |  |  | Shelter | | Org (120+ h) | Y |
| Lafollette 2019 | ✓ | ✓ | ✓ | ✓ |  | ✓ | Shelter; Rescue; Agency transfer; Other | | Org (60+ h) | N |
| McLaughlin 2019 | - | | | | | | - | | - | - |
| Scotland-Coogan 2019a | - | | | | | | - | | V (-) | - |
| Scotland-Coogan 2019b | - | | | | | | - | | V (-) | - |
| Whitworth 2019 | - | | | | | | Shelter; Rescue | | V (14 w) | Y |
| Crowe 2018a | - | | | | | | Shelter | | V (up to 12 m) | Y |
| Crowe 2018b | - | | | | | | Shelter | | V (up to 18 m) | Y |
| Lessard 2018 | ✓ | ✓ |  |  |  | ✓ | - | | Org (-) | Y |
| O'Haire 2018 | ✓ | ✓ |  |  | ✓ |  | Shelter | | - | Y |
| Rodriguez 2018 | - | | | | | | Shelter | | Org (120+ h) | Y |
| Yarborough 2018 | - | | | | | | - | | - | Y |
| Kloep 2017 |  | ✓ | ✓ |  |  |  | Purpose-bred | | Org (8-12 m) | Y |
| Vincent 2017b | - | | | | | | - | | - | Y |
| Yarborough 2017 | - | | | | | | - | | Org (-) | Y |
| Dissertation |  | | | | | |  | |  |  |
| Floore-Guetschow 2020 | - | | | | | | - | | Org; Self-trained; Correctional facility (-) | - |
| Hansen 2019 | - | | | | | | - | | - | Y |
| Parenti 2019 | - | | | | | | - | | - | - |
| Kegel 2016 | - | | | | | | - | | - | - |
| Kopicki 2016 | - | | | | | | - | | - | - |
| Brown 2015 | - | | | | | | - | | - | - |
| Hyde 2015 | ✓ |  |  |  |  |  | Rescue; Purpose-bred | | Correctional facility (2 y) | - |
| Marston 2015 | - | | | | | | - | | - | - |
| Moore 2014 | - | | | | | | - | | Org (600+ h) | Y |
| Newton 2014 | - | | | | | | - | | - | Y |

***Notes.*** *Ordered by most recent to least recent within each group. - Not reported. LR Labrador Retriever. GR Golden Retriever. LX Labrador Retriever Mix. GSD German Shepherd Dog. X Mixed breed. V Veteran (with organizational guidance).*

*^a^ - Trained tasks are not reported; N Trained tasks are reported, but individual PTSD-specific tasks are not described; Y Trained tasks are reported, and PTSD-specific trained tasks are described.*
