# Supplementary material for: Assistance dogs for military veterans with PTSD: A systematic review, meta-analysis, and meta-synthesis
Source: PLoS One. 2022 Sep 21;17(9):e0274960. doi: 10.1371/journal.pone.0274960 (PMC9491613; doi:10.1371/journal.pone.0274960)
Supplement: Attachment — Submitted filename: ResponseToReviewers_220906.docx [file pone.0274960.s007.docx]

Dear Reviewers,

Thank you very much for your time and expertise in reviewing our manuscript.

**Reviewer #1:**

We were not provided with any comments from Reviewer #1. We thank them again for their time and expertise in reviewing our manuscript and hope that we were successful in addressing all of their previous comments.

**Reviewer #2:**

All comments have been addressed.

We are pleased to have addressed all of Reviewer #2’s comments and thank them again for their valuable feedback, which we believe has strengthened the quality of our manuscript.
